# Supplementary material for: Using machine learning to design a short test from a full-length test of functional health literacy in adults—The development of a short form of the Danish TOFHLA
Source: PLoS One. 2023 Jul 27;18(7):e0280613. doi: 10.1371/journal.pone.0280613 (PMC10373996; doi:10.1371/journal.pone.0280613)
Supplement: S1 Appendix — (PDF) [file pone.0280613.s001.pdf]

DS-TOFHLA – the Danish version

’Test of Functional Literacy in Adults’

’Test of Functional Literacy in Adults’

TOFHLA

’Reading comprehension’

Instruction

- Hand patient the reading comprehension passages to be completed. Make sure that the patient sees only the text.
- PREFACE THE READING COMPREHENSION EXERCISE WITH:

" Here are some other medical instructions that you or anybody might see around the hospital. These instructions are in sentences that have some of the words missing. Where a word is missing, a blank line is drawn, and 4 possible words that could go in the blank appear just below it. I want you to figure out which of those 4 words should go in the blank, which word makes the sentences make sense. When you think you know which one it is, circle the letter in front of that word, and go on to the next one. When you finish the page, turn the page and keep going until you finish all the pages"

STOP AFTER 5 MINUTTER

PASSAGE A: X-RAY PREPARATION

PASSAGE B: HEALTH INSURANCE "DENMARK" RIGHTS AND RESPONSIBILITIES

PASSAGE C: HOSPITAL CONSENT FORM

PASSAGE A

You must have an _____________ stomach when you come for _____________.

a. asthma | a. is.
b. empty | b. am.
c. incest | c. if.
d. anemia | d. it.

THE DAY OF THE X-RAY

Do not _____________, even _____________.

a. drive, | a. heart.
b. drink, | b. breath.
c. dress, | c. water.
d. dose, | d. cancer.

PASSAGE B

I ______________ to provide the health insurance 'Denmark' information that

a. agree
b. examine
c. send
d. gain

______________ all statements given in this ______________ and hereby give

a. hides
b. risks
c. discharges
d. proves

a. emphysema
b. application
c. gallbladder
d. relationship

permission to the ______________ to get such proof.

a. inflammation
b. religion
c. iron
d. health insurance

If I experience any ______________ in my circumstances, I must report these

a. changes
b. hormones
c. antacids
d. charges

______________ after becoming ______________ of the change.

a. within a week
b. within a month
c. within three months
d. immediately

a. award
b. aware
c. away
d. await

AFSNIT C

It has been explained to _____________ that during the course of the

a. my

b. me

c. he

d. she

_____________ or procedure, unforeseen conditions may be _____________

a. syphilis	a. revealed
b. hepatitis	b. depressed
c. colitis	c. directed
d. operation	d. notified

that necessitate an extension of the _____________ procedure(s) or different

a. appendix

b. another

c. original

d. addict

procedure(s) than those _____________ forth in clinical guidelines.

a. get

b. set

c. see

d. go

I, therefore, _____________ and request that the above named _____________,

a. exercise
b. authorize
c. energize
d. pressurize

a. infection
b. pregnant
c. insurance
d. physician

his assistants or attending physicians _____________ such procedures as are

a. perform
b. smear
c. onset
d. stress

necessary and ______________ in the exercise of professional judgement.

a. undesirable
b. emergency
c. desirable
d. diagnosis

THANK YOU FOR YOUR REPLY
